# Integrated untargeted and targeted metabolomics identifies and validates urinary bile acid biomarkers for the diagnosis of intrahepatic cholestasis of pregnancy

**DOI:** 10.3389/fendo.2026.1944962

**Published:** 2026-09-23

**Authors:** Haiyue You, Jingyi Gao, Xin Ning, Feng Zhang, Yida Wang, Xinfeng Yang, Yan Zhang, Jianfeng Lu, Jingying Jiang

**Affiliations:** 1 Department of Obstetrics, Wuxi Maternity and Child Health Care Hospital, Women’s Hospital of Jiangnan University, Jiangnan University, Wuxi, China; 2 Suzhou Medical College, Soochow University, Suzhou, Jiangsu, China; 3 Department of Obstetrics, Wuxi Maternity and Child Health Care Hospital, Wuxi Medical Center, Nanjing Medical University, Wuxi, China

**Keywords:** bile acid biomarkers, cholic acid, glycocholic acid, intrahepatic cholestasis of pregnancy, targeted metabolomics, taurocholic acid, untargeted metabolomics

## Abstract

**Background:**

Intrahepatic cholestasis of pregnancy (ICP) is the most common pregnancy-specific liver disorder and is characterized by unexplained pruritus and elevated serum bile acid levels, typically in the third trimester. Excessive bile acid accumulation is associated with maternal discomfort and adverse fetal outcomes, underscoring the need for timely and accurate diagnosis.

**Methods:**

We enrolled 76 pregnant women. In the discovery cohort, untargeted metabolomics was performed on paired serum and urine samples from 8 patients with ICP and 8 healthy controls to identify co-differentially expressed metabolites (co-DEMs). In the validation cohort, targeted metabolomics was used to quantify urinary bile acid co-DEMs in 30 patients with ICP and 30 controls.

**Results:**

Untargeted metabolomics identified 76 and 64 differentially expressed metabolites (DEMs) in serum and urine, respectively. Seven co-DEMs were detected in both sample types, five of which were bile acid species. Kyoto Encyclopedia of Genes and Genomes (KEGG) enrichment analysis highlighted primary bile acid biosynthesis as a key pathway associated with ICP. Targeted metabolomics showed marked increases in urinary glycocholic acid (GCA) and taurocholic acid (TCA), both of which remained significant after false discovery rate (FDR) correction, whereas cholic acid (CA) showed a more modest difference in the candidate-focused analysis. Urinary concentrations of these metabolites were positively correlated with serum total bile acid levels. Receiver operating characteristic (ROC) curve analysis showed strong diagnostic performance for GCA (AUC = 0.95) and TCA (AUC = 0.86), whereas CA showed modest performance (AUC = 0.65).

**Conclusion:**

Primary bile acid biosynthesis is closely associated with ICP. Urinary GCA and TCA may serve as promising non-invasive biomarkers for the diagnosis of ICP. These findings require confirmation in larger, independent multicenter cohorts before clinical implementation.

## Background

Intrahepatic cholestasis of pregnancy (ICP) is the most common liver disorder during pregnancy and is clinically characterized by elevated serum bile acid levels, usually in the third trimester, together with otherwise unexplained pruritus, particularly on the palms and soles (1). A clinical diagnosis of ICP should be considered when a pregnant woman presents with pruritus and normal-appearing skin, together with a fasting total bile acid (TBA) concentration greater than 10 μmol/L or a peak random TBA concentration of 19 μmol/L or higher (2, 3). ICP is associated with severe adverse pregnancy outcomes, including preterm birth, intrauterine asphyxia, meconium-stained amniotic fluid, and fetal bradycardia (4, 5). The risk of fetal complications increases with increasing maternal serum bile acid levels, particularly when concentrations reach 40 μmol/L or higher (6). A recent meta-analysis reported an increased risk of stillbirth in singleton pregnancies complicated by ICP when serum bile acid concentrations were 100 μmol/L or higher (5). Therefore, timely diagnosis and effective intervention are essential for maternal and fetal health (2, 3, 7). However, although TBA is routinely used in clinical practice, it may not fully meet the need for more precise diagnosis and risk stratification (8–10). Identifying novel biomarkers with diagnostic, prognostic, or therapeutic relevance may help address these clinical challenges (8).

Maternal metabolism undergoes substantial changes during pregnancy (11, 12). Bile acid metabolism contributes to metabolic homeostasis by facilitating intestinal nutrient absorption and biliary cholesterol secretion, and is tightly regulated through hepatic bile acid synthesis and intestinal bile acid biotransformation (13, 14). However, excessive accumulation of bile acids in the liver or other organs can disrupt this balance and contribute to metabolic disorders, with ICP representing a pregnancy-specific example (11, 12). Pregnancy itself has been associated with mild increases in bile acid concentrations (11). Physiological hypercholanemia during pregnancy usually has no adverse maternal or fetal effects, whereas pathologically elevated serum bile acid levels can impair homeostasis and contribute to ICP (12). Given the central role of bile acid metabolism in ICP pathogenesis, profiling bile acid species may provide an effective approach for identifying potential diagnostic biomarkers (8, 15, 16).

Metabolomics is a powerful approach for biomarker discovery because it enables the comprehensive profiling of metabolites in biofluids, cells, and tissues (17). Two complementary strategies are commonly used: untargeted and targeted metabolomics. Untargeted metabolomics enables the simultaneous detection of hundreds to thousands of metabolites and is characterized by broad metabolite coverage, high throughput, and high resolution (18, 19). In contrast, targeted metabolomics measures predefined panels of biochemically annotated metabolites and provides confident identification, high analytical precision, and accurate quantification (19, 20). Therefore, integrating untargeted and targeted metabolomics may strengthen the discovery and validation of biomarkers with high sensitivity and specificity for ICP diagnosis (8, 19). In this study, we integrated untargeted and targeted metabolomics to screen and validate potential biomarkers for ICP. First, untargeted metabolomics identified seven co-DEMs detected concurrently in serum and urine samples. We then performed targeted bile acid profiling in an expanded cohort to quantify candidate metabolites. Finally, urinary glycocholic acid and taurocholic acid showed strong diagnostic potential as non-invasive biomarkers for ICP. The novelty of the present study lies not in the first identification of GCA or TCA as ICP-associated metabolites, but in the use of paired serum–urine untargeted metabolomics to prioritize concordantly altered co-DEMs, followed by quantitative validation of urinary bile acid candidates in a separate expanded cohort. This cross-biofluid discovery strategy differs from previous studies focused primarily on urine alone, urinary sulfated bile acids, or circulating bile acid panels.

## Materials and methods

### Patients and samples

Two clinical cohorts comprising 76 pregnant women were included in this study. All participants were recruited from Wuxi Maternal and Child Health Care Hospital between September 2021 and June 2022. The study was approved by the Research Ethics Committee of Wuxi Maternal and Child Health Care Hospital (Institutional Review Board approval number: 2020-01-0310-08), and all participants provided written informed consent. The discovery cohort included 8 pregnant women with ICP and 8 healthy pregnant controls, whereas the validation cohort included 30 pregnant women with ICP and 30 healthy pregnant controls. The discovery cohort was designed for exploratory untargeted metabolomics and hypothesis generation, while the larger validation cohort was used for targeted quantitative assessment of the candidate urinary bile acid biomarkers. No formal *a priori* sample-size calculation was performed for the exploratory discovery stage. The validation cohort was expanded to 30 participants per group to improve the robustness of subsequent biomarker evaluation.

ICP was diagnosed based on the presence of: 1) unexplained maternal pruritus, predominantly involving the palms and soles and not attributable to other causes; 2) liver dysfunction, reflected by elevated serum aspartate aminotransferase (AST) and alanine aminotransferase (ALT) levels; and 3) elevated fasting serum bile acid levels (≥10 μmol/L). Patients with abnormal liver function due to other conditions that could increase bile acid levels were excluded, including viral hepatitis, preeclampsia, HELLP syndrome, primary biliary cholangitis, and acute fatty liver of pregnancy.

Serum and urine samples from all participants in the discovery cohort were collected for untargeted metabolomics. Given the non-invasive and convenient nature of urine testing, particularly in pregnant women, urine samples from all participants in the validation cohort were collected for subsequent targeted bile acid metabolomics. Most samples were collected 1–2 weeks before the expected date of delivery, whereas samples from women who delivered preterm were collected on the day of delivery. All serum and urine samples were stored at -80 °C until metabolite extraction.

### Metabolite extraction

For untargeted metabolomics, metabolites were extracted from serum and urine samples as follows. Briefly, 100 μL of each sample was mixed with 300 μL of ice-cold 80% aqueous methanol in centrifuge tubes, vortexed thoroughly, and sonicated at 4 °C for 30 min. The samples were then incubated at -20 °C for 60 min and centrifuged at 12,000 rpm for 15 min at 4 °C. Next, 200 μL of supernatant was mixed with 5 μL of the internal standard 2-chloro-1-phenylalanine (GL Biochem, Shanghai, China) and transferred to LC vials. Quality control (QC) samples were prepared by pooling 10 μL aliquots of extract from each specimen. QC samples were analyzed before, during, and after the analytical sequence. The pre-run QC samples were used to assess instrument status and equilibrate the chromatographic–mass spectrometric system, whereas QC samples inserted during the analytical sequence were used to monitor system stability.

For targeted metabolomics of urine samples, stock standard solutions were prepared by dissolving standards in methanol at a concentration of 1 mg/mL. Working solutions 1 and 2 were then prepared by serial dilution of the stock solution. Working solution 1 was used to prepare mixed calibration standards, and working solution 2 was used to optimize mass spectrometry conditions. The concentrations of the mixed calibration standards were 100, 50, 25, 12.5, 6.25, 3.12, 1.56, 0.78, 0.39, 0.19, 0.09, and 0.04 ng/mL. For each sample, 200 μL of urine was mixed with 800 μL of ice-cold acetonitrile containing 5% ammonia and two isotopic internal standards, bile acid-2,2,4,4-d4 and glycocholic acid-(glycine-1-13C). The mixture was vortexed for 30 s, extracted by ultrasonication in ice water for 20 min, and incubated at -20 °C for 30 min. After centrifugation at 13,000 rpm for 10 min at 4 °C, 900 μL of supernatant was evaporated to dryness and reconstituted in 200 μL of methanol/water solution (1:1, v/v) containing the internal standards L-2-chlorophenylalanine and Lyso PC17:0. The solution was vortexed for 30 s, sonicated for 5 min, and centrifuged at 13,000 rpm for 10 min at 4 °C. Finally, 200 μL of supernatant was collected with a syringe, filtered through a 0.22 μm organic-phase syringe filter, and stored in amber vials at -80 °C until analysis. QC samples were prepared by pooling 10 μL aliquots of extract from each sample.

### Untargeted metabolomics profiling and data analysis

Liquid chromatography–mass spectrometry (LC-MS; Waters UPLC; Thermo Q Exactive) was used for untargeted metabolomic profiling of serum and urine samples. The detailed chromatographic and mass spectrometric procedures and detection parameters have been described in our previous study and are provided in the **Supplementary File** (21). After raw data acquisition, individual peaks were filtered by retaining features with no more than 50% missing values within a single group or across all groups. Remaining missing values were imputed using one-half of the minimum detected value, and the data were normalized to the total ion current (TIC) of each sample. After preprocessing, 6023 and 4170 molecular features were retained in the positive- and negative-ion modes, respectively, for serum samples, whereas 6129 and 6219 features were retained in the corresponding positive- and negative-ion modes for urine samples. The processed data were analyzed using SIMCA software (version 14.1; Sartorius Stedim Data Analytics AB) for multivariate analyses, including principal component analysis (PCA), partial least squares-discriminant analysis (PLS-DA), and orthogonal partial least squares-discriminant analysis (OPLS-DA) (22). Before supervised multivariate modeling, the data were log-transformed and UV-scaled. The PLS-DA and OPLS-DA models were evaluated using seven-fold cross-validation. Model robustness and potential overfitting were further assessed using permutation testing with 200 permutations. Metabolites with a variable importance in projection (VIP) value >1 and a nominal *P* value <0.05 were initially considered differentially expressed metabolites (DEMs) in the exploratory untargeted metabolomics analysis (21). DEMs detected in both serum and urine samples were defined as co-differentially expressed metabolites (co-DEMs). To account for multiple comparisons, *P* values for all tested features were additionally adjusted using the Benjamini-Hochberg false discovery rate (FDR) procedure within each ionization mode. FDR-adjusted *P* values were used as a sensitivity analysis to assess the robustness of the exploratory findings.

Hierarchical clustering was performed to compare the expression patterns of DEMs in serum and urine samples, and Z-score plots were generated to visualize the relative abundance of DEMs. Pearson correlation coefficients were calculated to assess correlations among DEMs. Kyoto Encyclopedia of Genes and Genomes (KEGG) pathway enrichment analysis was performed using the R package clusterProfiler (version 4.6.0). KEGG metabolic pathways and associated metabolites were downloaded from the KEGG REST API. DEMs were also mapped to authoritative metabolite databases, including KEGG, the Human Metabolome Database (HMDB), and PubChem, for metabolite annotation. Fold enrichment and mean log2 fold change were calculated for DEMs within each pathway to characterize and rank enriched KEGG pathways.

### Targeted metabolomics profiling and data analysis

Liquid chromatography–tandem mass spectrometry (LC-MS/MS) was performed using an AB ExionLC system coupled to an AB Sciex Qtrap 6500+ mass spectrometer (AB Sciex) for targeted profiling of urinary bile acid species. Detailed chromatographic and mass spectrometric parameters are provided in the **Supplementary File**. During spectral analysis, automatic identification and integration of each multiple reaction monitoring transition were performed using the default parameters in SCIEX OS-MQ software (Sciex, USA), followed by manual inspection. Overlay analysis of TIC plots from QC samples was performed to evaluate the reproducibility of metabolite detection. Based on retention time and peak shape, the mass spectrometry peaks of each metabolite were manually calibrated across samples to ensure qualitative and quantitative accuracy. Peak areas were used to represent the relative abundance of each metabolite, and peak-area integration results were obtained for all samples. After preparation and serial dilution of mixed standard solutions, quantitative mass spectrometry data were acquired at different standard concentrations and used to generate standard curves. The regression equation was as follows (x-axis: standard concentration; y-axis: mass spectrometry peak area):


$$y\;=\;0.00853\;x\;+\;0.01433\left({\text{r}}^{2}=\;0.99908,\text{ weighting}:\;1/\text{x}\right)$$

Metabolite peak areas were substituted into the regression equation fitted to the standard curve to calculate metabolite concentrations. The absolute content of each metabolite in actual samples was then calculated according to the conversion formula provided in the **Supplementary File**. Quantitative metabolite data were analyzed to obtain the mean metabolite concentration in each group and fold change between groups. Between-group differences were assessed using Welch’s t-test. To account for multiple comparisons across the targeted bile acid panel, *P* values were adjusted using the Benjamini–Hochberg false discovery rate (FDR) procedure. Bile acid species with an FDR-adjusted *P* value <0.05 were considered statistically significant. Urinary bile acid concentrations were reported as absolute concentrations (ng/mL) and were not normalized to urinary creatinine.

By integrating co-DEMs with differentially expressed bile acid species, we identified bile acid species co-DEMs and obtained their quantitative concentrations. Pearson correlation analysis was then performed to evaluate associations between urinary concentrations of bile acid species co-DEMs and serum clinical indicators, including TBA, AST, ALT, and alkaline phosphatase (ALP). The 95% confidence intervals (CIs) for Pearson correlation coefficients were calculated using Fisher’s z transformation. A correlation coefficient greater than 0.5 was considered to indicate a strong correlation between the metabolite and the clinical indicator. Receiver operating characteristic (ROC) curves were generated to assess the diagnostic performance of bile acid species co-DEMs as potential biomarkers for ICP. ROC curves based on binary logistic regression were also generated to evaluate the diagnostic value of combined biomarkers consisting of more than one metabolite. Cutoff values, sensitivity, specificity, Youden index, and area under the curve (AUC) were calculated from the ROC curves. The 95% confidence intervals of AUCs were calculated for each ROC curve. An AUC of 0.8-0.9 was considered to indicate excellent diagnostic discrimination, whereas an AUC greater than 0.9 was considered outstanding (23).

### Statistical analysis

GraphPad Prism 9.0 and R 4.2.3 were used for statistical analysis and data visualization. Specifically, Pearson correlation and ROC analyses, together with their corresponding visualizations, were performed using GraphPad Prism 9.0. Student’s t-test was used for normally distributed data, and the Mann-Whitney U test was used for non-normally distributed data. Qualitative, quantitative, and statistical methods specific to untargeted and targeted metabolomics are described in the corresponding Methods subsections and figure legends. A two-sided *P* value <0.05 was considered statistically significant (*P < 0.05, **P < 0.01, ***P < 0.001, and ****P < 0.0001).

## Results

### Overview of the discovery cohort and validation cohort

The overall study design is shown in **Figure 1**. A total of 76 participants were enrolled and assigned to the discovery cohort or validation cohort. The baseline characteristics of healthy pregnant women and patients with ICP in the two cohorts are summarized in **Table 1**. Compared with healthy pregnant women (CON group), pregnant women diagnosed with ICP (ICP group) had higher levels of TBA, AST, ALT, and ALP and delivered at an earlier gestational age.

**Figure 1:**
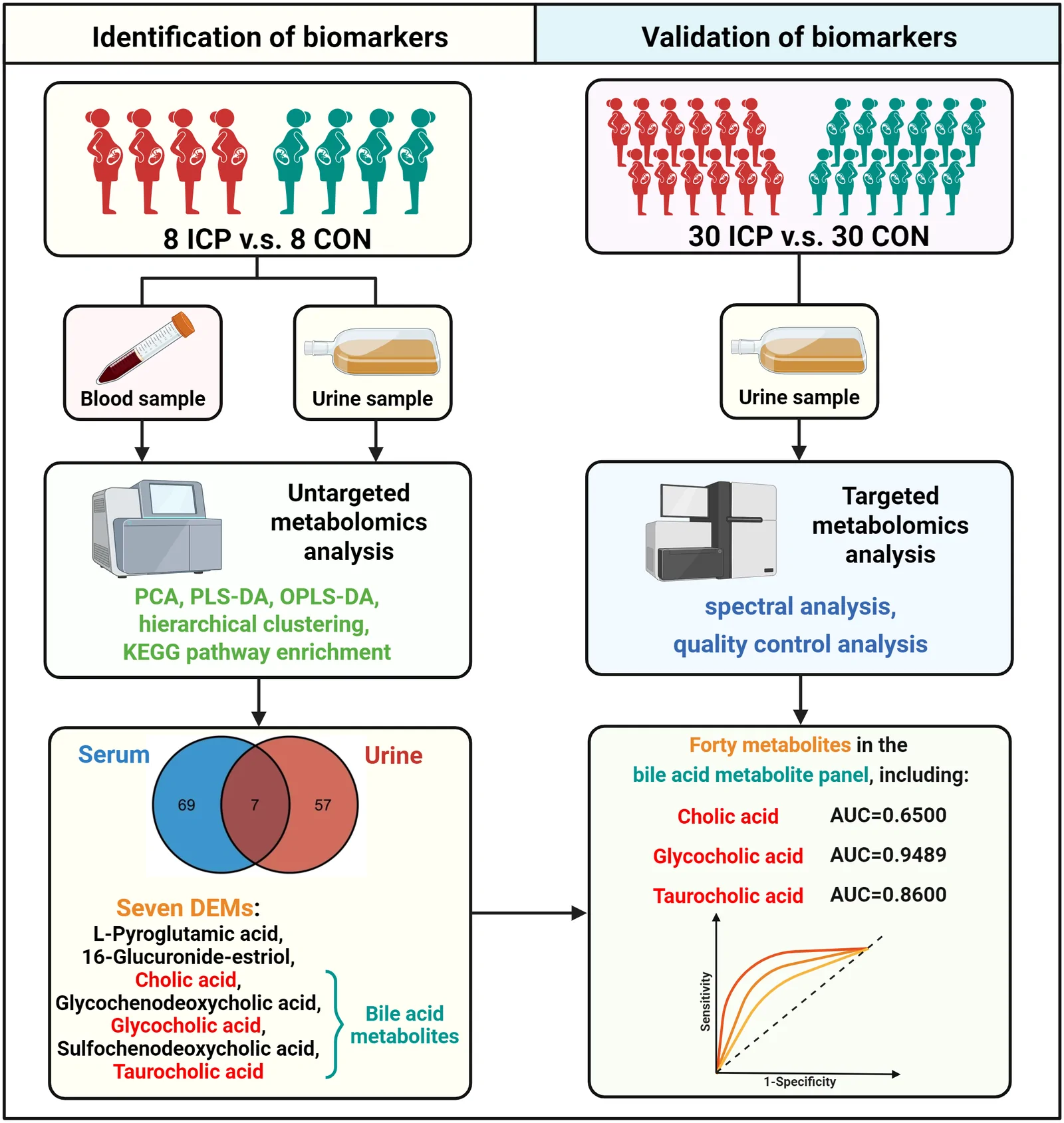
The graphical overview of study design and main findings.

**Table 1:** Baseline characteristics of healthy pregnant women and patients with ICP in the discovery and validation cohorts.

	Discovery cohort			Validation cohort		
	CON group (n=8)	ICP group (n=8)	*P* value	CON group (n=30)	ICP group (n=30)	*P* value
Age (year)			0.6917			0.5435
Mean ± SD.	32.4±4.9	31.4±5.0		31.5 ± 4.8	30.8 ± 4.5	
Median(IQR)	32.5(28.5-36.3)	31.5(26.8-35.5)		30.5(27.8-34.0)	31.0(26.8-34.3)	
Range	25.0-40.0	24.0-39.0		25.0-44.0	23.0-39.0	
BMI (kg/m^2^)			0.3383			0.4467
Mean ± SD.	28.3 ± 2.9	26.7 ± 3.6		26.9 ± 3.7	26.3 ± 2.7	
Median (IQR)	29.0(25.9-29.4)	27.8(24.4-28.4)		26.3(24.5-28.9)	26.3(24.7-28.3)	
Range	23.4-32.9	19.5-31.3		20.6-36.7	19.5-31.3	
TBA (μmol/L)			0.0025			<0.0001
Mean ± SD.	2.7 ± 0.8	65.2± 48.2		3.7 ± 2.1	42.2 ± 32.3	
Median (IQR)	2.7(1.9-3.4)	50.7(28.7-87.1)		3.4(2.2-4.5)	34.1(19.2-54.7)	
Range	1.4-3.8	20.4-166.0		1.3-8.9	10.1-166.0	
AST (U/L)			0.0020			<0.0001
Mean ± SD.	9.1 ± 1.6	101.9 ± 69.5		14.6 ± 7.8	82.7 ± 77.0	
Median (IQR)	9.3(8.1-10.2)	108.9(36.8-145.1)		11.7(9.1-16.0)	66.3(9.1-143.9)	
Range	6.3-11.4	12.9-212.1		6.3-37.8	3.3-243.9	
ALT (U/L)			0.0025			<0.0001
Mean ± SD.	16.4 ± 3.4	67.8 ±39.4		19.1 ± 4.2	60.5± 54.3	
Median (IQR)	16.6(13.2-19.6)	59.0(33.6-110.6)		19.1(16.3-21.0)	44.2(17.8-70.3)	
Range	11.0-20.4	30.1-130.4		11.0-27.9	10.8-235.0	
ALP (U/L)			0.0075			0.0133
Mean ± SD.	153.7 ± 65.2	236.1 ± 36.2		167.0 ± 65.5	213.1 ± 74.1	
Median (IQR)	136.1(107.7-181.1)	236.7(212.5-262.9)		151.7(118.6-193.0)	217.4(159.8-251.1)	
Range	100.1-296.6	175.1-292.6		88.8-367.0	81.9-407.2	
Gestational age at delivery (days)			<0.0001			<0.0001
Mean ± SD.	274.0 ± 2.7	258.9 ± 7.5		276.0 ± 4.9	262.4 ± 10.6	
Median (IQR)	273.0(272.3-276.3)	259.5(253.3-266.0)		274.0(273.0-279.0)	262.0(256.3-267.5)	
Range	271.0-279.0	246.0-267.0		268.0-286.0	240.0-282.0	
Gravidity (no.)			0.4570			0.6336
Mean ± SD.	2.6 ± 1.4	3.3 ± 1.8		2.1 ± 1.2	2.2 ± 1.5	
Median (IQR)	2.5(1.3-3.8)	3.0(2.0-4.0)		2.0(1.0-3.0)	2.0(1.0-3.0)	
Range	1.0-5.0	1.0-7.0		1.0-5.0	1.0-7.0	
Parity (no.)			0.9999			0.6669
Mean ± SD.	0.8 ± 0.7	0.8 ± 0.7		0.4 ± 0.6	0.5 ± 0.6	
Median (IQR)	1.0(0.0-1.0)	1.0(0.0-1.0)		0.0(0.0-1.0)	0.0(0.0-1.0)	
Range	0.0-2.0	0.0-2.0		0.0-2.0	0.0-2.0	

1) Student’s t-test was used when variances were homogeneous; Welch’s t-test was used when variances were heterogeneous.

2) CON, healthy control; ICP, intrahepatic cholestasis of pregnancy; SD, standard deviation; IQR, interquartile range; BMI, body mass index; TBA, total bile acid; AST, aspartate aminotransferase; ALT, alanine aminotransferase; ALP, alkaline phosphatase.

In the untargeted metabolomics analysis, 6023 and 4170 features were retained in positive- and negative-ion modes, respectively, for serum samples, and 6129 and 6219 features were retained for urine samples. PCA models were subsequently constructed based on the processed molecular features. QC samples clustered closely, indicating satisfactory analytical stability (**Figures 2A, B**; **Supplementary Figures 1A, B**). Because PCA is an unsupervised method and does not use group labels, supervised models were further applied. Both PLS-DA and OPLS-DA clearly separated the ICP group from the CON group in serum samples (**Figures 2C, E**) and urine samples (**Figures 2D, F**). The PLS-DA and OPLS-DA models were evaluated using seven-fold cross-validation and 200-permutation testing. For the urine dataset, the PLS-DA model yielded R²X(cum) = 0.36, R²Y(cum) = 0.97, and Q²(cum) = 0.846, whereas the OPLS-DA model yielded R²X(cum) = 0.36, R²Y(cum) = 0.97, and Q²(cum) = 0.718. In the permutation analyses, the permuted Q² values were lower than those of the corresponding original models, and the Q² regression-line intercepts were below zero, supporting model robustness and indicating no evident overfitting (**Supplementary Figures 1C–F**). Similarly, spectral analysis of the targeted metabolomics data indicated good instrument stability, reproducibility, and quality control (**Supplementary Figure 2**).

**Figure 2:**
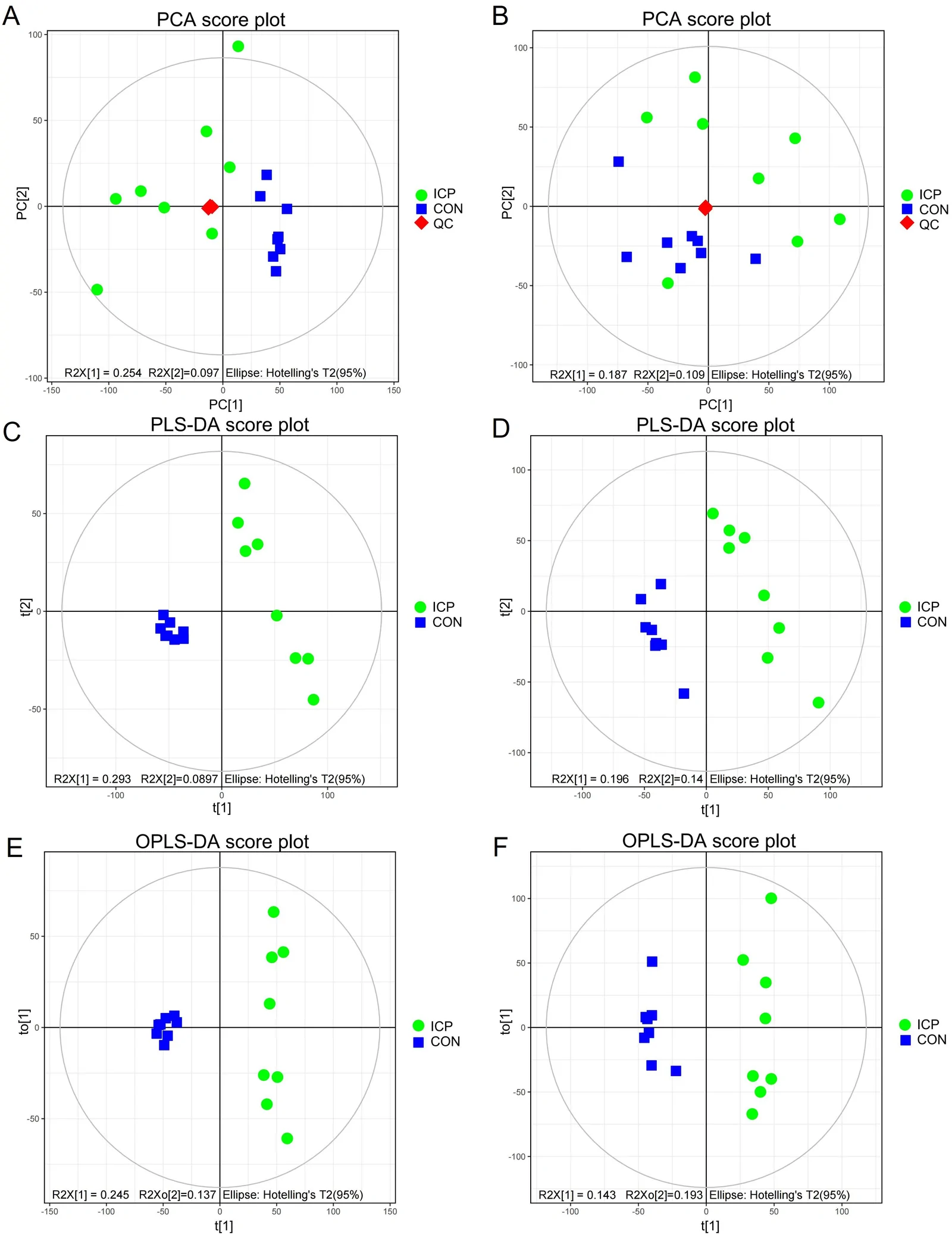
PCA, PLS-DA, and OPLS-DA score plots from the untargeted metabolomics analysis. **(A, B)** PCA score plots of serum **(A)** and urine **(B)** samples from patients with ICP (green), healthy controls (blue), and QC samples (red). **(C, D)** PLS-DA score plots of serum **(C)** and urine **(D)** samples from patients with ICP and healthy controls. **(E, F)** OPLS-DA score plots of serum **(E)** and urine **(F)** samples from patients with ICP and healthy controls. Hotelling’s T² (95%) ellipses define the statistical boundary within which 95% of samples in each group are expected to fall under multivariate normality and highlight potential metabolic outliers. PCA, principal component analysis; PLS-DA, partial least squares-discriminant analysis; OPLS-DA, orthogonal partial least-squares-discriminant analysis; ICP, intrahepatic cholestasis of pregnancy; CON, healthy control; QC, quality control.

### Preliminary screening of DEMs through untargeted metabolomics

Volcano plots were generated for metabolites in serum (**Figure 3A**) and urine (**Figure 3B**) samples. Using VIP >1 and a nominal *P* value <0.05 as the exploratory screening criteria, we identified 76 DEMs in serum samples (**Table 2**) and 64 DEMs in urine samples (**Table 3**). Benjamini–Hochberg FDR correction was additionally performed across all tested features within each ionization mode to evaluate the robustness of these findings. After feature-level Benjamini–Hochberg FDR correction, 35 of the 76 serum DEMs remained significant, whereas none of the 64 urinary DEMs remained significant. Among the seven co-DEMs, GCA and TCA did not meet the FDR threshold in the discovery cohort. Therefore, the untargeted metabolomics results were considered exploratory and hypothesis-generating, and the candidate bile acids were subsequently evaluated in the expanded targeted validation cohort. Among the 76 serum DEMs, 35 were upregulated and 41 were downregulated in the ICP group (**Figure 3C**). Among the 64 urine DEMs, 13 were upregulated and 51 were downregulated in patients with ICP (**Figure 3D**). Sulfochenodeoxycholic acid, 3-methylcrotonylglycine, and taurocholic acid were the three most upregulated DEMs in serum samples, whereas sulfochenodeoxycholic acid, cholic acid, and acetylproline were the three most upregulated DEMs in urine samples. LysoPE(20:5), isoleucylisoleucine, and itaconate were the three most downregulated DEMs in serum samples, whereas nonanoic acid, cyclo-prolylglycine, and mono-iso-butylphthalate were the three most downregulated DEMs in urine samples. Sulfochenodeoxycholic acid showed the largest fold increase among the nominally screened DEMs in both serum and urine samples, highlighting it as a candidate metabolite warranting further targeted validation. Z-scores, calculated from relative metabolite abundances, are shown in **Supplementary Figure 3**. DEMs in serum samples showed clearer separation between ICP and control groups than DEMs in urine samples. To assess concordant trends between serum and urine DEMs, we generated metabolite correlation heatmaps (**Figure 4**) and examined associations among metabolites using Pearson correlation coefficients. Substantial correlations were observed among most DEMs in both serum (**Figure 4A**) and urine (**Figure 4B**) samples, suggesting coordinated metabolic alterations associated with ICP. These correlation patterns illustrate coordinated metabolic alterations and provide additional context for subsequent biomarker selection and pathway analysis.

**Figure 3:**
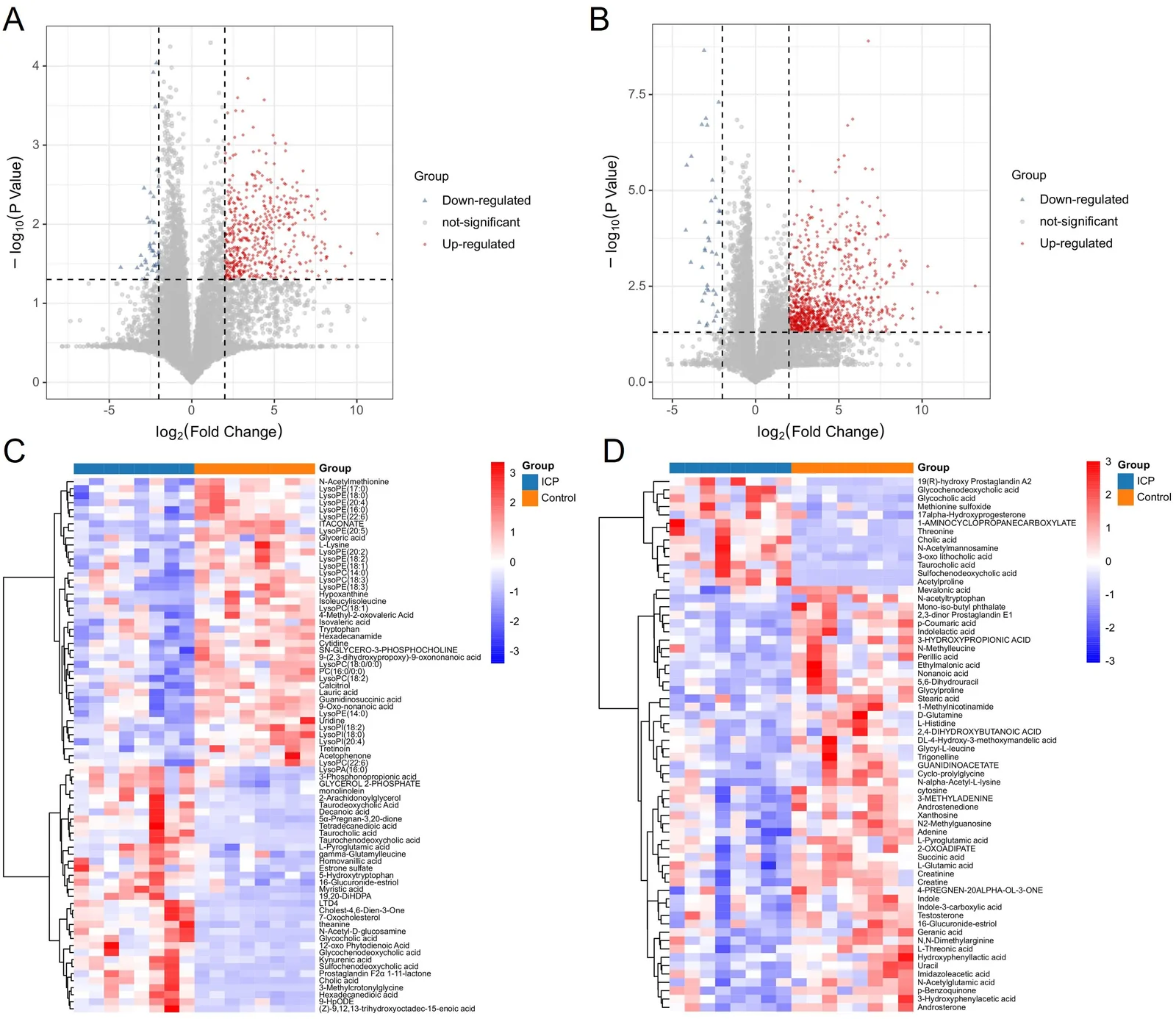
Preliminary screening of DEMs by untargeted metabolomics. **(A, B)** Volcano plots showing DEMs in serum **(A)** and urine **(B)** samples between patients with ICP and healthy controls. **(C, D)** Clustering heatmaps of DEMs in serum **(C)** and urine **(D)** samples between patients with ICP and healthy controls. The color scale represents Z-score-normalized relative metabolite abundance. DEMs, differentially expressed metabolites; ICP, intrahepatic cholestasis of pregnancy.

**Table 2:** Differentially expressed metabolites in serum samples (merged positive- and negative-ion modes).

Metabolite name	Formula	Molecular weight	m/z	VIP	*P* value	Fold change
Sulfochenodeoxycholic acid	C24 H40 O7 S	472.24518	471.23767	1.978576751	0.002786877	680.1613329
3-Methylcrotonylglycine	C7 H11 N O3	157.07284	158.08012	1.895276208	0.011362622	85.21732783
Taurocholic acid	C26 H45 N O7 S	515.28796	516.29547	1.699873577	0.045199237	44.21668015
Taurochenodeoxycholic acid	C26 H45 N O6 S	499.29245	498.28503	1.71018413	0.036427501	15.51982279
Glycochenodeoxycholic acid	C26 H43 N O5	449.30971	448.30215	1.659249954	0.033207464	14.41006001
Glycocholic acid	C26 H43 N O6	465.30577	466.31308	1.683392059	0.015676445	10.25019039
N-Acetyl-D-glucosamine	C8 H15 N O6	221.08847	222.09569	1.499785864	0.015618954	9.639806645
Cholic acid	C24 H40 O5	408.28393	407.27643	1.698544145	0.015782591	8.915850715
2-Arachidonoylglycerol	C23 H38 O4	378.27205	379.27933	1.528012276	0.046672254	6.268875736
Taurodeoxycholic Acid	C26 H45 N O6 S	499.2932	500.30054	1.496726809	0.044537716	5.999042599
LTD4	C25 H40 N2 O6 S	496.26347	495.2562	1.811716911	0.001750615	5.385083449
7-Oxocholesterol	C27 H44 O2	400.33136	401.33862	1.731700311	0.010711293	5.223029898
Cholest-4,6-Dien-3-One	C27 H42 O	382.3209	383.32816	1.607510611	0.026678269	4.902549481
theanine	C7 H14 N2 O3	174.10329	175.11057	1.751737231	0.003357343	4.21195339
5α-Pregnan-3,20-dione	C21 H32 O2	316.23796	317.24521	1.644681716	0.00705437	4.118577823
12-oxo Phytodienoic Acid	C18 H28 O3	292.20193	293.20901	1.377057027	0.026371324	3.822309919
Tetradecanedioic acid	C14 H26 O4	258.18093	257.17355	1.769694641	0.018806831	3.762176654
19,20-DiHDPA	C22 H34 O4	362.2429	361.23563	1.626152268	0.006197529	3.654750256
(Z)-9,12,13-trihydroxyoctadec-15-enoic acid	C18 H34 O5	330.23798	329.23071	1.600153478	0.034477917	3.567682756
Hexadecanedioic acid	C16 H30 O4	286.21215	285.2048	1.716257959	0.003609763	3.161231989
Kynurenic acid	C10 H7 N O3	189.04036	188.03305	1.632663581	0.012046164	2.91417746
9-HpODE	C18 H32 O4	312.22755	311.22025	1.328726992	0.018570387	2.75170391
LysoPA(16:0)	C19 H39 O7 P	410.24005	409.23282	1.294115038	0.005949669	2.750039235
Estrone sulfate	C18 H22 O5 S	350.11593	349.10846	1.154748035	0.024552186	2.258883991
Prostaglandin F2α 1-11-lactone	C20 H32 O4	336.22737	335.22009	1.474956814	0.011643511	2.189230943
monolinolein	C21 H38 O4	354.27476	355.28207	1.293486605	0.010442247	2.007505836
3-Phosphonopropionic acid	C3 H7 O5 P	154.00204	155.00932	1.436328536	0.003081337	1.997363734
Decanoic acid	C10 H20 O2	172.14397	171.13664	1.164407382	0.045414846	1.940938495
16-Glucuronide-estriol	C24 H32 O9	464.201	463.19366	1.354199146	0.007196486	1.88390141
Homovanillic acid	C9 H10 O4	182.05554	181.04826	1.154669594	0.027870466	1.712159254
Myristic acid	C14 H28 O2	228.2067	227.19939	1.342917361	0.012549112	1.70329965
5-Hydroxytryptophan	C11 H12 N2 O3	220.08255	219.07521	1.473627031	0.002561992	1.615303604
GLYCEROL 2-PHOSPHATE	C3 H9 O6 P	172.01262	173.01997	1.108578052	0.029261727	1.574855909
gamma-Glutamylleucine	C11 H20 N2 O5	260.13505	259.12769	1.183806118	0.034276335	1.350036624
L-Pyroglutamic acid	C5 H7 N O3	129.04188	130.04918	1.17021621	0.022274555	1.344025725
Isovaleric acid	C5 H10 O2	102.06785	103.0752	1.016965613	0.045042369	0.856164082
SN-GLYCERO-3-PHOSPHOCHOLINE	C8 H20 N O6 P	257.10103	258.10837	1.364858051	0.003244177	0.840875787
Tryptophan	C11 H12 N2 O2	204.08862	205.09576	1.029998963	0.039591297	0.826776389
Hexadecanamide	C16 H33 N O	255.2544	256.26187	1.389387224	0.006530638	0.825281167
LysoPC(18:0/0:0)	C26 H54 N O7 P	523.36011	524.36743	1.061471179	0.033300732	0.816854998
Lauric acid	C12 H24 O2	200.17533	199.16774	1.308631458	0.012199692	0.808089669
LysoPC(18:1)	C26 H52 N O7 P	521.34445	522.35156	1.224500278	0.0242767	0.798995154
LysoPE(14:0)	C19 H40 N O7 P	425.24996	424.24332	1.572854475	0.000819117	0.784477022
Cytidine	C9 H13 N3 O5	243.08736	242.08005	1.437590277	0.003042255	0.768687344
LysoPE(18:0)	C23 H48 N O7 P	481.31276	480.3053	1.048768618	0.018912268	0.762306267
9-Oxo-nonanoic acid	C9 H16 O3	172.10769	171.10049	1.564480799	0.000453552	0.757477006
LysoPE(18:1)	C23 H46 N O7 P	479.29704	478.28961	1.139111427	0.027903095	0.752448706
4-Methyl-2-oxovaleric Acid	C6 H10 O3	130.06074	129.05347	1.215780681	0.012564777	0.743174281
LysoPE(17:0)	C22 H46 N O7 P	467.29727	466.29001	1.069985901	0.017363539	0.737852323
9-(2,3-dihydroxypropoxy)-9-oxononanoic acid	C12 H22 O6	262.13945	261.13226	1.719759955	2.72221E-05	0.733592514
LysoPC(22:6)	C30 H50 N O7 P	567.3281	568.33551	1.116751699	0.026865875	0.727039331
LysoPE(16:0)	C21 H44 N O7 P	453.28117	452.27383	1.070704307	0.01601391	0.726262538
L-Lysine	C6 H14 N2 O2	146.10458	147.11179	1.170133817	0.036757326	0.725666309
LysoPC(18:2)	C26 H50 N O7 P	519.32851	520.33569	1.510538721	0.000782155	0.689612162
PC(16:0/0:0)	C24 H50 N O7 P	495.32819	496.33566	1.63220615	9.3446E-05	0.684279233
LysoPE(20:4)	C25 H44 N O7 P	501.28147	500.27435	1.480026943	0.001389766	0.673220364
LysoPC(18:3)	C26 H48 N O7 P	517.31334	518.32074	1.105007211	0.026733799	0.667912665
LysoPC(14:0)	C22 H46 N O7 P	467.29799	468.30527	1.043488347	0.040806971	0.661156274
Uridine	C9 H12 N2 O6	244.06726	243.06003	1.409107086	0.006853868	0.654705225
LysoPE(18:2)	C23 H44 N O7 P	477.2814	476.27399	1.422367612	0.003705116	0.637505154
Guanidinosuccinic acid	C5 H9 N3 O4	175.05742	176.06473	1.556280099	0.000220482	0.634283411
LysoPE(22:6)	C27 H44 N O7 P	525.28113	524.27386	1.505901695	0.000210308	0.611521537
LysoPI(20:4)	C29 H49 O12 P	620.29101	619.28418	1.673610422	0.000188715	0.607710548
LysoPI(18:2)	C27 H49 O12 P	596.29131	595.28412	1.348153429	0.013647134	0.605766174
Calcitriol	C27 H44 O3	416.32582	415.31857	1.077450684	0.017184013	0.598658783
LysoPE(20:2)	C25 H48 N O7 P	505.31296	504.30582	1.451841848	0.001509598	0.587149611
Glyceric acid	C3 H6 O4	106.02453	105.01723	1.150946001	0.015993629	0.569547805
LysoPE(18:3)	C23 H42 N O7 P	475.26609	474.25888	1.269476551	0.010057449	0.556511472
Tretinoin	C20 H28 O2	300.2065	299.19919	1.234491565	0.011978049	0.544270498
LysoPI(18:0)	C27 H53 O12 P	600.32297	599.31592	1.625418657	0.004431511	0.542784149
N-Acetylmethionine	C7 H13 N O3 S	191.05935	190.05208	1.189006573	0.005076552	0.495525723
Hypoxanthine	C5 H4 N4 O	136.03766	137.04488	1.361146831	0.010181918	0.494336107
Acetophenone	C8 H8 O	120.05691	121.06419	1.224559832	0.035435157	0.470984251
ITACONATE	C5 H6 O4	130.02434	129.01709	1.328235282	0.003707107	0.423550609
Isoleucylisoleucine	C12 H24 N2 O3	244.17652	243.16919	1.284678642	0.030397761	0.396908252
LysoPE(20:5)	C25 H42 N O7 P	499.26605	498.25885	1.383970976	0.003051682	0.375906031

1) Differentially expressed metabolites were initially screened using VIP >1 and a nominal *P* value <0.05 in the exploratory untargeted metabolomics analysis. Benjamini–Hochberg FDR correction was additionally applied to all tested features within each ionization mode as a sensitivity analysis for multiple comparisons;

2) Student’s t-test was used to calculate statistical significance.

3) Abbreviations and explanations:

Formula, molecular or predicted chemical formula obtained from qualitative matching analysis;

Molecular weight, measured molecular weight of the detected substance; m/z, mass-to-charge ratio of a characteristic ion; VIP, variable importance in projection;

Fold change, ratio of mean(ICP)/mean(CON); CON, control group; ICP, intrahepatic cholestasis of pregnancy group.

Red text indicates metabolites that are upregulated (increased) in the ICP group compared with the CON group, with a Fold change (ICP/CON) > 1. Blue text indicates metabolites that are downregulated (decreased) in the ICP group compared with the CON group, with a Fold change (ICP/CON) < 1.

**Table 3:** Differentially expressed metabolites in urine samples (merged positive- and negative-ion modes).

Metabolite name	Formula	Molecular weight	m/z	VIP	*P* value	Fold change
Sulfochenodeoxycholic acid	C24 H40 O7 S	472.24502	471.23773	2.491710344	0.007351779	234.689826
Cholic acid	C24 H40 O5	408.28426	407.27695	2.283554651	0.007397243	53.36902449
Acetylproline	C7 H11 N O3	157.07279	158.08006	2.516094512	0.000945203	33.03516392
Glycochenodeoxycholic acid	C26 H43 N O5	449.31052	448.30316	1.457121351	0.043428838	8.117555073
Taurocholic acid	C26 H45 N O7 S	515.28725	514.27997	1.671038431	0.042520982	7.73348504
N-Acetylmannosamine	C8 H15 N O6	221.08837	222.09566	1.843245486	0.012346612	7.630290735
3-oxo lithocholic acid	C24 H38 O3	374.27941	375.28693	2.310539151	0.001350725	5.681488781
19(R)-hydroxy Prostaglandin A2	C20 H30 O5	350.20682	351.21384	1.686632717	0.035275828	3.848619506
Glycocholic acid	C26 H43 N O6	465.3052	464.29791	1.829712024	0.007244259	3.667817546
Threonine	C4 H9 N O3	119.05793	120.06491	1.774631013	0.021817128	3.665290553
1-AMINOCYCLOPROPANECARBOXYLATE	C4 H7 N O2	101.04759	102.05477	1.635504035	0.0449271	2.723622052
17alpha-Hydroxyprogesterone	C21 H30 O3	330.21713	331.22446	1.643621442	0.033527141	2.441483591
Methionine sulfoxide	C5 H11 N O3 S	165.04486	166.05214	1.517387648	0.015758604	1.377620193
L-Glutamic acid	C5 H9 N O4	147.05085	146.04358	1.418533116	0.027711949	0.835157236
Indole-3-carboxylic acid	C9 H7 N O2	161.04661	162.05377	1.425232526	0.024501204	0.760054204
2-OXOADIPATE	C6 H8 O5	160.03489	159.02759	1.4004849	0.030068381	0.734920461
Adenine	C5 H5 N5	135.0537	136.06104	1.484964823	0.00666378	0.727924744
Succinic acid	C4 H6 O4	118.0244	117.01717	1.285936487	0.049709361	0.71902591
2,4-DIHYDROXYBUTANOIC ACID	C4 H8 O4	120.04314	121.05031	1.288966123	0.041934741	0.710306492
cytosine	C4 H5 N3 O	111.04282	112.0501	1.302361526	0.038577239	0.708162148
Indole	C8 H7 N	117.05732	118.06461	1.380237358	0.031493193	0.699571789
N-Acetylglutamic acid	C7 H11 N O5	189.06145	188.05414	1.376889994	0.046335411	0.696863048
N-alpha-Acetyl-L-lysine	C8 H16 N2 O3	188.1147	189.12221	1.671344303	0.019908405	0.69569763
L-Pyroglutamic acid	C5 H7 N O3	129.0403	128.03305	1.490159085	0.020483807	0.677480927
Xanthosine	C10 H12 N4 O6	284.07341	283.06619	1.362171537	0.041189563	0.668377088
L-Threonic acid	C4 H8 O5	136.0349	135.02762	1.79481011	0.001821038	0.666391035
3-METHYLADENINE	C6 H7 N5	149.06912	150.07631	1.572653184	0.011407147	0.664738
Testosterone	C19 H28 O2	288.2069	289.2142	1.526040114	0.049571331	0.653520824
N,N-Dimethylarginine	C8 H18 N4 O2	202.14175	203.14896	1.778345023	0.009725913	0.641333103
Creatine	C4 H9 N3 O2	131.06894	132.07603	1.598762127	0.008732623	0.639323115
Creatinine	C4 H7 N3 O	113.05846	114.06574	1.751019938	0.001713805	0.635674996
N2-Methylguanosine	C11 H15 N5 O5	297.10486	296.0976	1.613131253	0.012910319	0.626150243
Glycylproline	C7 H12 N2 O3	172.08366	173.09105	1.776146953	0.012373333	0.618654926
Mevalonic acid	C6 H12 O4	148.07119	147.06395	1.408281852	0.012075668	0.615379121
Stearic acid	C18 H36 O2	284.26927	283.26208	1.411807542	0.045017516	0.606726635
L-Histidine	C6 H9 N3 O2	155.06719	154.05991	1.530624559	0.029371081	0.605497409
3-HYDROXYPROPIONIC ACID	C3 H6 O3	90.03188	91.03896	1.494134	0.033112479	0.604296845
4-PREGNEN-20ALPHA-OL-3-ONE	C21 H32 O2	316.23805	317.24533	1.311009914	0.044534858	0.598348718
Geranic acid	C10 H16 O2	168.11274	167.10545	1.583399366	0.039021748	0.598275738
GUANIDINOACETATE	C3 H7 N3 O2	117.05314	118.06068	1.581750043	0.020833028	0.585103038
Indolelactic acid	C11 H11 N O3	205.07262	206.07997	1.573067269	0.0151292	0.575508904
Imidazoleacetic acid	C5 H6 N2 O2	126.04071	125.03345	1.543192999	0.040126501	0.550454147
3-Hydroxyphenylacetic acid	C8 H8 O3	152.04628	153.05363	1.657025355	0.014060638	0.548849448
DL-4-Hydroxy-3-methoxymandelic acid	C9 H10 O5	198.05056	197.0433	1.440307448	0.042570797	0.54265694
N-Methylleucine	C7 H15 N O2	145.10803	144.10078	1.679883147	0.030598171	0.541065004
p-Benzoquinone	C6 H4 O2	108.02078	109.02799	1.570408698	0.016784352	0.53956102
Ethylmalonic acid	C5 H8 O4	132.04002	131.03275	1.599701947	0.032661925	0.494575186
16-Glucuronide-estriol	C24 H32 O9	464.20052	463.1933	1.425431411	0.044404064	0.48558637
5,6-Dihydrouracil	C4 H6 N2 O2	114.04252	115.04982	2.174877786	0.00116772	0.470317015
1-Methylnicotinamide	C7 H8 N2 O	136.0628	137.07008	1.311255523	0.035406699	0.464995515
Perillic acid	C10 H14 O2	166.09716	165.08986	1.699042699	0.010089842	0.451410261
D-Glutamine	C5 H10 N2 O3	146.06688	145.05959	1.809888279	0.018695849	0.451149835
Androstenedione	C19 H26 O2	286.19132	287.19855	1.833242342	0.00400718	0.437935591
Glycyl-L-leucine	C8 H16 N2 O3	188.11377	187.10649	1.678505731	0.017568655	0.427482304
2,3-dinor Prostaglandin E1	C18 H30 O5	326.2068	325.19955	2.174605327	0.000104842	0.427174107
Androsterone	C19 H30 O2	290.22261	291.22983	1.693681992	0.007729965	0.417166849
Trigonelline	C7 H7 N O2	137.04678	138.05408	1.742662425	0.041443613	0.406296006
Uracil	C4 H4 N2 O2	112.02507	111.01778	1.789990509	0.003038966	0.383978388
N-acetyltryptophan	C13 H14 N2 O3	246.09829	245.09099	1.252377669	0.028247796	0.349613943
Hydroxyphenyllactic acid	C9 H10 O4	182.05563	181.04832	1.613022937	0.026687244	0.339533281
p-Coumaric acid	C9 H8 O3	164.0463	165.05367	1.755340455	0.000692973	0.321572215
Mono-iso-butylphthalate	C12 H14 O4	222.0877	223.09491	1.523954961	0.040100881	0.309295665
Cyclo-prolylglycine	C7 H10 N2 O2	154.07324	155.08044	1.714972723	0.027322597	0.275424887
Nonanoic acid	C9 H18 O2	158.12835	157.12109	1.759012419	0.032983944	0.273218525

1) Differentially expressed metabolites were initially screened using VIP >1 and a nominal P value <0.05 in the exploratory untargeted metabolomics analysis. Benjamini–Hochberg FDR correction was additionally applied to all tested features within each ionization mode as a sensitivity analysis for multiple comparisons;

2) Student’s t-test was used to calculate statistical significance.

3) Abbreviations and explanations:

Formula, molecular or predicted chemical formula obtained from qualitative matching analysis;

Molecular weight, measured molecular weight of the detected substance; m/z, mass-to-charge ratio of a characteristic ion; VIP, variable importance in projection; Fold change, ratio of mean(ICP)/mean(CON); CON, control group; ICP, intrahepatic cholestasis of pregnancy group.

Red text indicates metabolites that are upregulated (increased) in the ICP group compared with the CON group, with a Fold change (ICP/CON) > 1. Blue text indicates metabolites that are downregulated (decreased) in the ICP group compared with the CON group, with a Fold change (ICP/CON) < 1.

**Figure 4:**
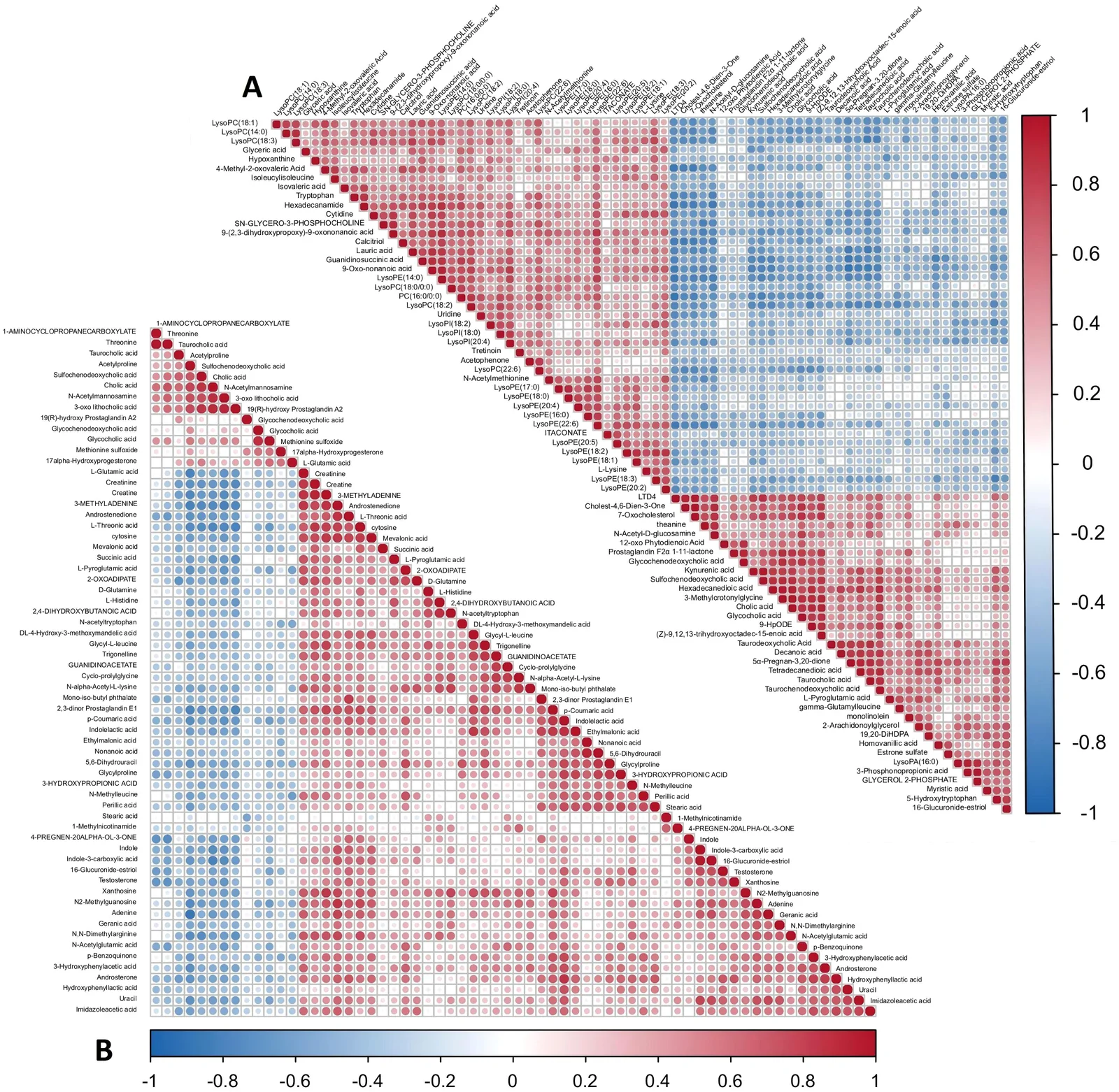
Correlation heatmaps of DEMs in serum **(A)** and urine **(B)** samples from patients with ICP and healthy controls. Pearson correlation coefficients among DEMs ranged from -0.65 to +0.92 in serum samples **(A)** and from -0.71 to +0.89 in urine samples **(B)**. Color intensity represents the strength and direction of correlation, with red indicating positive correlation and blue indicating negative correlation.

### Identification of co-DEMs in serum and urine samples

To explore metabolic pathways associated with DEMs, KEGG pathway enrichment analysis was performed. In serum samples, primary bile acid biosynthesis was the only top enriched metabolic pathway (**Figure 5A**; **Supplementary Table 1**). In urine samples, the top enriched metabolic pathways included β-alanine metabolism, histidine metabolism, D-glutamine and D-glutamate metabolism, and primary bile acid biosynthesis (**Figure 5B**, **Supplementary Table 1**). Notably, primary bile acid biosynthesis was enriched in both serum and urine samples, suggesting that this pathway may play an important role in ICP. By comparing DEMs in serum and urine samples, we identified seven co-DEMs: L-pyroglutamic acid, 16-glucuronide-estriol, cholic acid, glycochenodeoxycholic acid, glycocholic acid, sulfochenodeoxycholic acid, and taurocholic acid (**Figure 5C**; **Table 4**). Among these, cholic acid, glycochenodeoxycholic acid, glycocholic acid, sulfochenodeoxycholic acid, and taurocholic acid were bile acid species co-DEMs, and all were upregulated in the ICP group in both serum and urine samples. In contrast, L-pyroglutamic acid and 16-glucuronide-estriol showed opposite trends, with higher levels in serum samples and lower levels in urine samples from the ICP group (**Table 4**). These findings support a pivotal role for bile acid species in ICP pathogenesis and suggest that bile acid alterations are consistently reflected at both serum and urinary levels.

**Figure 5:**
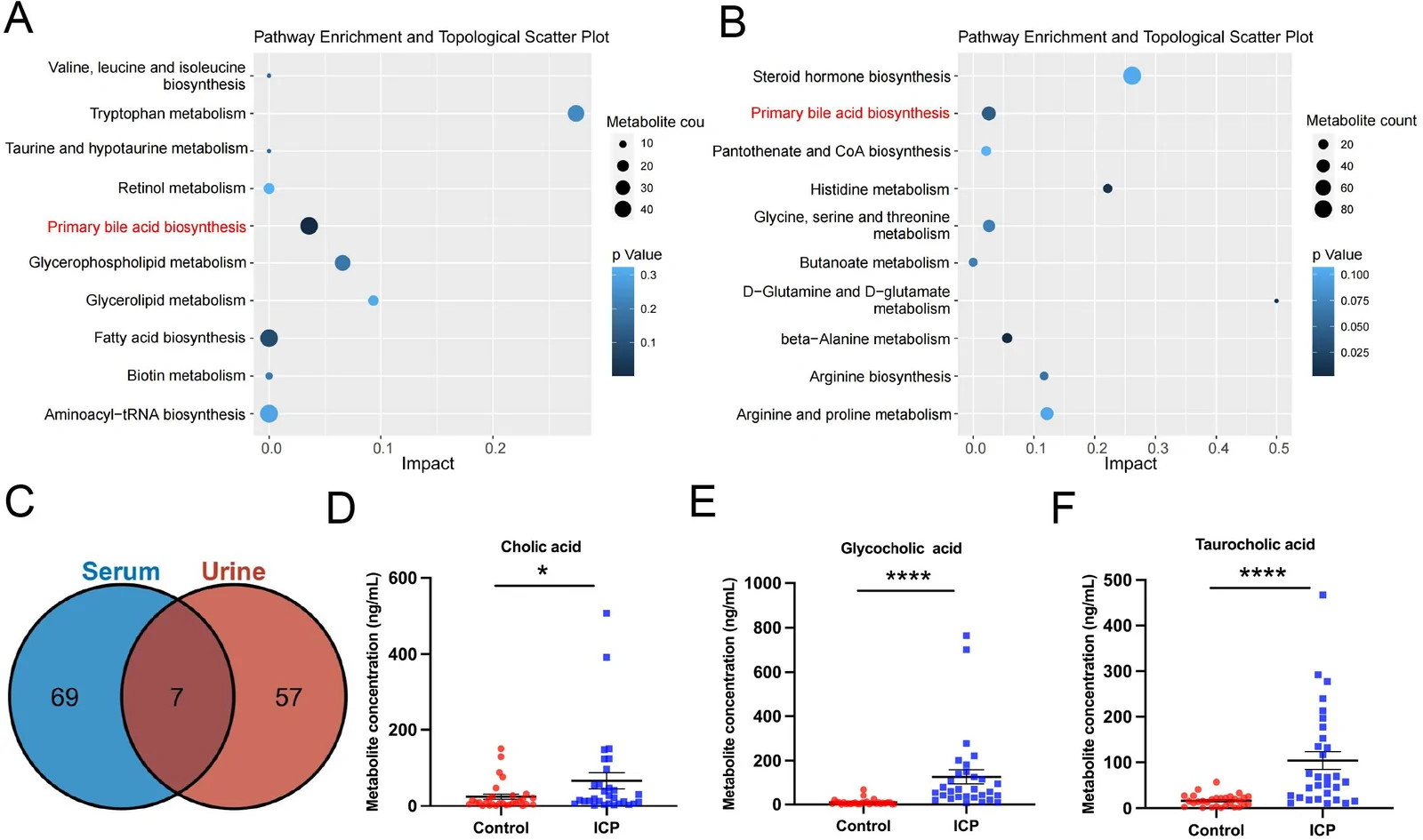
Identification of primary bile acid biosynthesis as a key pathway and quantitative analysis of three bile acid species co-DEMs. **(A, B)** KEGG pathway analysis of DEMs in serum **(A)** and urine **(B)** samples. **(C)** Venn diagram showing co-DEMs shared between serum and urine samples. **(D-F)** Urinary levels of three bile acid species co-DEMs: cholic acid **(D)**, glycocholic acid **(E)**, and taurocholic acid **(F)**, in samples from the ICP and control groups. Data are presented as mean ± SEM. *P < 0.05, **P < 0.01, ***P < 0.001, and ****P < 0.0001; two-sided Mann-Whitney U test. KEGG, Kyoto Encyclopedia of Genes and Genomes; co-DEMs, co-differentially expressed metabolites.

**Table 4:** Co-differentially expressed metabolites detected in both serum and urine samples.

Metabolite name	Serum			Urine		
	Fold change (ICP/CON)	*P* value	Trend	Fold change (ICP/CON)	*P* value	Trend
L-Pyroglutamic acid	1.344025725	0.022274555	Up	0.677480927	0.020483807	Down
Cholic acid	8.915850715	0.015782591	Up	53.36902449	0.007397243	Up
Glycochenodeoxycholic acid	14.41006001	0.033207464	Up	8.117555073	0.043428838	Up
16-Glucuronide-estriol	1.88390141	0.007196486	Up	0.48558637	0.044404064	Down
Glycocholic acid	10.25019039	0.015676445	Up	3.667817546	0.007244259	Up
Sulfochenodeoxycholic acid	680.1613329	0.002786877	Up	234.689826	0.007351779	Up
Taurocholic acid	44.21668015	0.045199237	Up	7.73348504	0.042520982	Up

Co-DEMs were defined based on the exploratory untargeted metabolomics screening criteria (VIP >1 and nominal *P* < 0.05) in both serum and urine. FDR-adjusted results are reported separately in the Results.

### Validation of three co-DEMs in targeted bile acid species metabolomics

Given the critical role of bile acid metabolism in ICP, we recruited an expanded validation cohort of 30 patients with ICP and 30 healthy pregnant controls. To quantify the concentrations of bile acid species co-DEMs, targeted bile acid metabolomics was performed using a predefined panel of 40 bile acid species. Among the five bile acid species co-DEMs identified in the discovery analysis, cholic acid, glycocholic acid, and taurocholic acid were included in this targeted panel. Among the 31 bile acid species with valid statistical comparisons, 14 remained significantly different between the ICP and control groups after Benjamini-Hochberg FDR correction (FDR-adjusted *P* < 0.05) (**Table 5**). Notably, GCA and TCA remained significant after multiple-testing correction, with FDR-adjusted P values of 0.0038 and 0.0007, respectively. We then focused on the three bile acid species co-DEMs identified in the discovery analysis. GCA and TCA concentrations were markedly increased in the ICP group, with fold-change values of 12.70 and 6.27, respectively, and both remained significant after FDR correction. In the separate candidate-focused comparisons shown in **Figures 5D–F**, between-group differences were assessed using the Mann-Whitney U test; CA showed a more modest nominally significant increase, whereas GCA and TCA showed pronounced differences between groups.

**Table 5:** Differentially expressed bile acid species identified by targeted bile acid metabolomics.

Metabolite name	Ave (CON)/(ng/mL)	Ave (ICP)/(ng/mL)	Fold change (ICP/CON)	*P* value	FDR-adjusted *P* value
Glycoursodeoxycholic Acid	23.99057575	1201.976010	50.10200764	6.74951E-05	0.000749
β-Muricholic Acid	0.147631900	4.977765372	33.71741056	0.000576036	0.002232
Ursodeoxycholic Acid	3.608824736	89.02376333	24.66835323	9.48009E-05	0.000749
Tauroursodeoxycholic Acid	7.779483789	125.9958072	16.19590845	0.003469192	0.008962
Glycocholic Acid	9.998441678	126.9992002	12.70189938	0.001218164	0.003776
Taurohyocholic Acid	5.808785679	72.32939647	12.45172407	0.002429970	0.006848
Taurocholic Acid	16.65850436	104.4289487	6.268806995	0.000118146	0.000749
Chenodeoxycholic Acid Glycine Conjugate	5.157343348	30.33123706	5.881174671	0.000749779	0.002583
Taurochenodesoxycholic Acid	2.413088645	10.49821472	4.350530073	0.000248853	0.001286
Chenodeoxycholic Acid	0.822795865	3.153212767	3.832314795	0.015399338	0.034099
Glycohyocholic Acid	19.25776609	73.41281003	3.812114535	0.000382923	0.001696
Tauro-α-Muricholic Acid	60.27429357	184.0884411	3.054178326	8.33073E-05	0.000749
Tauro-β-Muricholic Acid (Sodium Salt)	60.78138236	184.9346575	3.042620130	0.000120748	0.000749
Hyocholic Acid	0.200439565	0.596030371	2.973616369	0.007854746	0.018731

1) Welch’s t-test was used to calculate statistical significance. *P* values were adjusted for multiple comparisons using the Benjamini–Hochberg false discovery rate (FDR) procedure.

2) Abbreviations or explanations: CON, control group; ICP, intrahepatic cholestasis of pregnancy group; Ave (ICP), average concentrations of metabolites in 30 urine specimens from pregnant women with ICP; Ave (CON), average concentrations of metabolites in 30 urine specimens from healthy pregnant women; Fold change, ratio of Ave (ICP)/Ave (CON).

### Clinical diagnostic values of three co-DEMs in the validation cohort

Pearson correlation analysis was performed to examine associations between urinary concentrations of bile acid species co-DEMs and serum clinical indicators (**Figures 6A–L**). Urinary concentrations of bile acid species co-DEMs showed generally positive trends with serum clinical indicators, with the strongest associations observed between GCA/TCA and serum TBA. In particular, urinary glycocholic acid and taurocholic acid concentrations were positively correlated with serum TBA levels (GCA: r = 0.6815, 95% CI: 0.517-0.797, *P* < 0.0001; TCA: r = 0.5072, 95% CI: 0.291-0.674, *P* < 0.0001) (**Figures 6B, C**). Urinary taurocholic acid was also positively correlated with serum ALP levels (r = 0.5474, 95% CI: 0.341-0.704, *P* < 0.0001) (**Figure 6L**). In addition, urinary cholic acid showed moderate positive correlations with serum TBA and ALT levels (r = 0.4696, 95% CI: 0.245-0.646, *P* = 0.0002; and r = 0.4117, 95% CI: 0.176-0.603, *P* = 0.0011, respectively) (**Figures 6A, G**).

**Figure 6:**
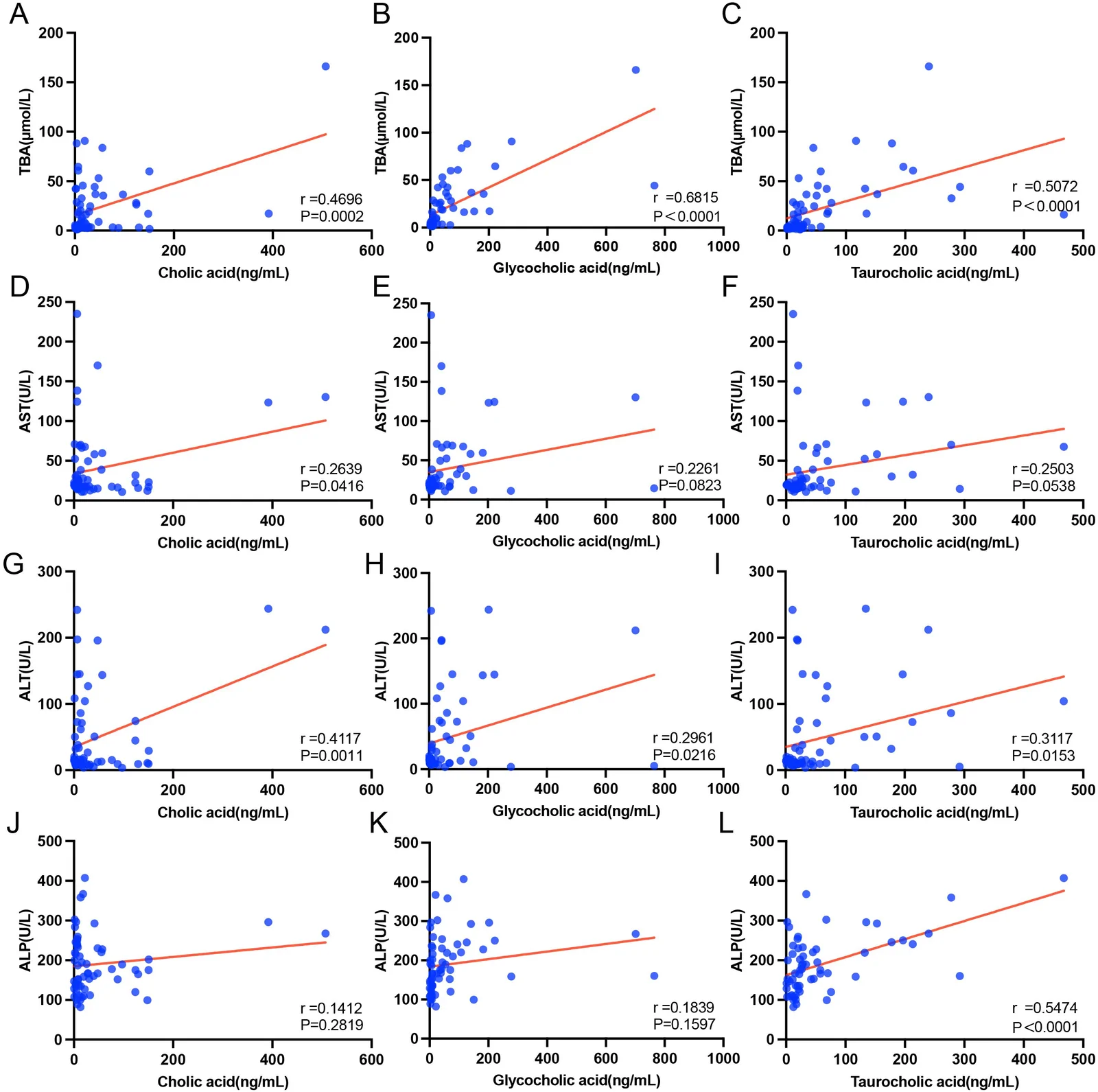
Correlation between three bile acid species co-DEMs and four clinical indicators (TBA, AST, ALT and ALP). **(A-C)** Correlations between TBA and urinary levels of cholic acid, glycocholic acid, and taurocholic acid. **(D–F)** Correlations between AST and urinary levels of cholic acid, glycocholic acid, and taurocholic acid. **(G–I)** Correlations between ALT and urinary levels of cholic acid, glycocholic acid, and taurocholic acid. **(J–L)** Correlations between ALP and urinary levels of cholic acid, glycocholic acid, and taurocholic acid.

We next evaluated the diagnostic potential of bile acid species co-DEMs in the validation cohort. ROC curves showed the diagnostic performance of cholic acid, glycocholic acid, taurocholic acid, and their combinations (**Figures 7A–G**). Glycocholic acid and taurocholic acid showed strong diagnostic performance, with AUC values of 0.9489 and 0.8600, respectively (**Figures 7B, C**), whereas cholic acid showed modest performance (AUC = 0.6500) (**Figure 7A**). Combinations containing glycocholic acid also effectively discriminated patients with ICP from healthy pregnant controls, with AUC values of approximately 0.95 (**Figures 7D, F**, G). The diagnostic utility of these biomarkers was further assessed in **Table 6**. To obtain the optimal Youden index, the cutoff values for cholic acid, glycocholic acid, and taurocholic acid were set at 13.00, 9.71, and 43.57 ng/mL, respectively. A glycocholic acid cutoff of 9.71 ng/mL yielded high sensitivity (0.9667), whereas a taurocholic acid cutoff of 43.57 ng/mL yielded high specificity (0.9667) (**Table 6**). These results support further evaluation of urinary glycocholic acid and taurocholic acid as candidate diagnostic biomarkers for ICP.

**Figure 7:**
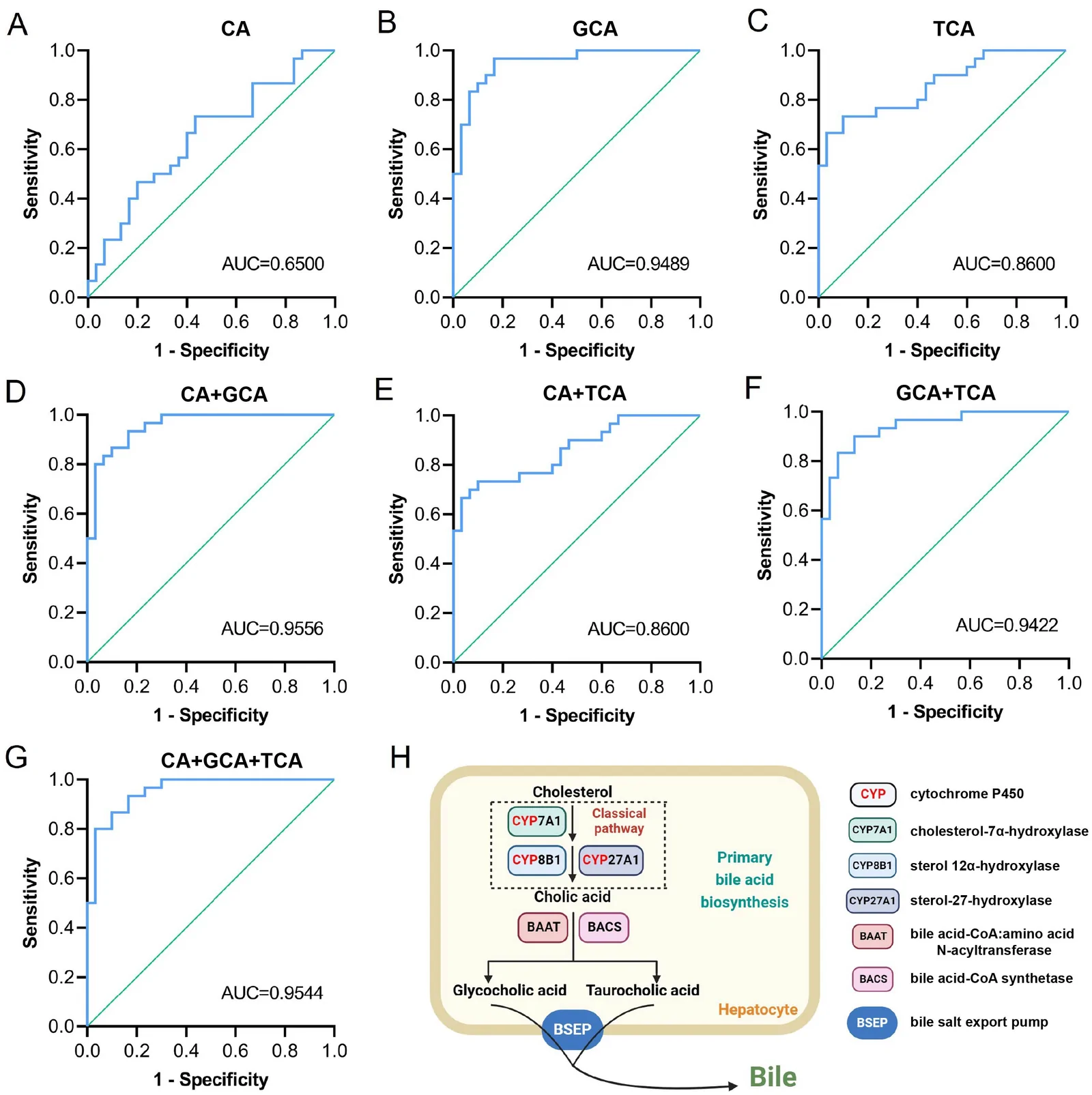
Single-marker and combined diagnostic performance of three bile acid species co-DEMs for identifying pregnant women with ICP. **(A–C)** ROC curves for cholic acid (CA), glycocholic acid (GCA), and taurocholic acid (TCA). **(D–G)** ROC curves for CA+GCA, CA+TCA, GCA+TCA, and CA+GCA+TCA. The AUCs (95% CIs) were 0.6500 (0.5107-0.7893) for CA, 0.9489 (0.8968-1.0000) for GCA, 0.8600 (0.7672-0.9528) for TCA, 0.9556 (0.9095-1.0000) for CA+GCA, 0.8600 (0.7671-0.9529) for CA+TCA, 0.9422 (0.8870-0.9974) for GCA+TCA, and 0.9544 (0.9078-1.0000) for CA+GCA+TCA. **(H)** Relationships among CA, GCA, and TCA in primary bile acid biosynthesis. CA, cholic acid; GCA, glycocholic acid; TCA, taurocholic acid; AUC, area under the receiver operating characteristic curve; CI, confidence interval; ICP, intrahepatic cholestasis of pregnancy; ROC, receiver operating characteristic. Combined biomarker models were constructed using binary logistic regression.

**Table 6:** Diagnostic utility of urinary bile acid species for identifying patients with ICP.

Biomarker	Cutoff value (ng/mL)	Diagnostic sensitivity	Diagnostic specificity	Youden index	AUC (95% CI)
CA	13.00	0.6667	0.6000	0.2667	0.6500 (0.5107-0.7893)
GCA	9.71	0.9667	0.8000	0.7667	0.9489 (0.8968-1.0000)
TCA	43.57	0.6667	0.9667	0.6334	0.8600 (0.7672-0.9528)
CA + GCA	NA	0.8667	0.9000	0.7667	0.9556 (0.9095-1.0000)
CA + TCA	NA	0.6667	0.9667	0.6334	0.8600 (0.7671-0.9529)
GCA + TCA	NA	0.9000	0.8667	0.7667	0.9422 (0.8870-0.9974)
CA + GCA + TCA	NA	0.8667	0.9000	0.7667	0.9544 (0.9078-1.0000)

CA, cholic acid; GCA, glycocholic acid; TCA, taurocholic acid; AUC, area under the receiver operating characteristic curve; CI, confidence interval; NA, not applicable. Combined biomarker models were constructed using binary logistic regression.

## Discussion

Although ICP is generally considered a self-limiting condition, it can have significant adverse effects on perinatal outcomes, highlighting the importance of early diagnosis and intervention (7, 24). Ursodeoxycholic acid (UDCA) is commonly used for ICP and can alleviate maternal pruritus and improve liver biochemical indices, although its effect on perinatal outcomes remains debated (9, 25). Early identification and intervention in patients with ICP are therefore clinically important (9, 10). Because TBA alone may not fully capture disease heterogeneity or diagnostic needs, additional biomarkers are needed to improve diagnostic precision in ICP management (8–10). In this study, we identified co-DEMs in both serum and urine samples and evaluated the diagnostic utility of three urinary bile acid species as non-invasive biomarkers for ICP.

Serum biomarkers directly reflect circulating metabolite levels, whereas urinary biomarkers offer non-invasive and convenient detection, which is particularly advantageous for pregnant women (8, 21, 26). Profiling paired serum and urine metabolites can help reveal underlying pathological processes, and urine-specific findings may provide complementary information related to renal handling and metabolite excretion (27). In the present study, we identified seven co-DEMs in both serum and urine samples, five of which were bile acid species that were elevated in patients with ICP. Their concordant alterations across both sample types support their relevance as cross-biofluid candidate markers.

Primary bile acid biosynthesis was the only pathway enriched in both sample types, highlighting the importance of bile acid metabolism in ICP pathogenesis (15, 16). Bile acids generally contain a steroidal structure, such as cholic acid, deoxycholic acid, chenodeoxycholic acid, or ursodeoxycholic acid, and participate in primary and secondary bile acid biosynthesis as well as enterohepatic circulation (14, 28–30). **Figure 7H** illustrates the relationships among cholic acid, glycocholic acid, and taurocholic acid in primary bile acid biosynthesis (14, 28, 31). Although the precise molecular mechanisms remain incompletely understood, disrupted bile acid metabolism is increasingly recognized as central to ICP pathogenesis and fetal development, providing a rationale for bile acid-based biomarker discovery (15, 16, 32). Recent evidence further suggests that gut microbiota-dependent remodeling of bile acid metabolism may contribute to ICP, while maternal-placental-fetal bile acid transport and sulfation also participate in bile acid homeostasis (30, 32). The relatively strong urinary signals of GCA and TCA may partly reflect differences in bile acid conjugation, transport, and renal excretion, although this interpretation remains speculative.

Among the three bile acid species examined in the validation cohort, urinary glycocholic acid and taurocholic acid were strongly correlated with serum TBA levels (r >0.5000, *P* < 0.0001) and showed strong diagnostic performance as urinary biomarkers. Consistent with our findings, previous studies have reported disrupted bile acid biosynthesis in association with elevated TBA levels in ICP based on untargeted metabolomics (33). Targeted metabolomics has also demonstrated the diagnostic potential of nine plasma bile acid species, including glycocholic acid (AUC = 0.994) and taurocholic acid (AUC = 1.000), in a cohort of 32 patients with ICP and 28 controls (31). These findings support the diagnostic relevance of bile acid species in ICP across different analytical methods and sample types (31, 33).

Targeted metabolomics enables quantitative measurement of metabolite concentrations and may facilitate clinical translation (19, 20). For example, urinary sulfated bile acids identified by high-performance LC-MS/MS were elevated in ICP and showed strong diagnostic potential in a cohort of 29 patients with ICP and 22 controls (26, 34). Reported biomarkers included cholic acid 3-sulfate (AUC = 0.873), glycine cholic acid 3-sulfate (AUC = 0.997), and taurine cholic acid 3-sulfate (AUC = 0.995) (26). Another study integrating proteomics and targeted metabolomics found elevated peroxisomal acyl-CoA oxidase 1 (ACOX1), L-palmitoylcarnitine, and glycocholic acid in serum and placenta and evaluated their diagnostic utility as serum biomarkers in a cohort of 40 patients with ICP and 40 controls, with AUC values of 0.823, 0.896, and 0.985, respectively; the combined biomarker panel achieved an AUC of 0.993 (35). These findings support the clinical feasibility of bile acid species metabolites, including sulfated forms, as potential diagnostic biomarkers across different sample types and further support the rationale of our study (26, 31, 35).

Although urinary sulfated bile acids have been investigated, many previous ICP biomarker studies have focused on circulating or placenta-related markers, which may limit broader clinical translation because of invasiveness, cost, or assay complexity (8, 26, 31, 33, 35). In contrast, this study integrated untargeted and targeted metabolomics using both serum and urine samples and identified five bile acid species co-DEMs showing concordant increases in both serum and urine. We further validated three urinary bile acid species as non-invasive and convenient candidate biomarkers for ICP diagnosis. This design extends prior biomarker studies that focused mainly on blood-based markers (8, 31, 33, 35).

This study has several limitations. First, the small discovery cohort may have limited statistical power and increased the risk of false-positive findings in the exploratory untargeted metabolomics analysis. Consistent with this limitation, none of the urinary DEMs remained significant after stringent feature-level FDR correction, underscoring the exploratory nature of the untargeted urinary findings and the importance of subsequent targeted validation in the expanded cohort. Second, the validation analysis was conducted in a single-center cohort with a relatively small sample size, and no additional internal resampling validation or independent external validation cohort was available. Therefore, the reported AUCs and cutoff values may be optimistic and require confirmation in larger, independent multicenter cohorts. Third, the cross-sectional design precludes evaluation of whether urinary bile acid species predict the future onset or worsening of ICP or subsequent perinatal outcomes. Fourth, the targeted bile acid panel did not include all five bile acid co-DEMs identified in the discovery analysis, particularly glycochenodeoxycholic acid and sulfochenodeoxycholic acid, and therefore full targeted validation of all discovery candidates was not possible. Fifth, detailed clinical and pre-analytical variables were not systematically collected, and residual confounding by these factors cannot be excluded. Sixth, quantitative LC-MS/MS measurements and the derived cutoff values may be platform- and laboratory-dependent, and assay harmonization will be required before clinical implementation. In addition, urinary bile acid concentrations were not normalized to urinary creatinine; therefore, interindividual variation in urine dilution may have influenced the absolute concentration estimates. Finally, this study did not investigate the mechanistic roles of bile acid species in ICP pathogenesis, particularly in relation to placental dysfunction (36). Future studies should validate these findings in larger, independent multicenter cohorts with standardized urine collection and normalization procedures and assess whether urinary GCA and TCA provide additional diagnostic value beyond conventional TBA testing. Prospective longitudinal and mechanistic studies are also needed to evaluate their potential value for disease monitoring and risk stratification and to clarify the biological basis of altered urinary bile acid excretion in ICP.

## Conclusion

By integrating untargeted and targeted metabolomics, we identified seven co-DEMs in paired serum and urine samples, highlighted the relevance of primary bile acid biosynthesis to ICP, and validated the diagnostic value of urinary bile acid species. Glycocholic acid and taurocholic acid showed high diagnostic performance and may serve as promising non-invasive biomarkers for ICP diagnosis.

## Data Availability

The original contributions presented in the study are included in the article/**Supplementary Material**. Further inquiries can be directed to the corresponding authors.

## Supplementary material

Supplementary Figure 1 **(A)** PCA box plots for serum samples, showing separation among patients with ICP, healthy controls, and QC samples. Kruskal-Wallis test P values indicate significant group differences for PC1 (*P* = 0.0013) and PC2 (*P* = 0.17). **(B)** PCA box plots for urine samples, showing separation among patients with ICP, healthy controls, and QC samples. Kruskal–Wallis test P values indicate group differences for PC1 (*P* = 0.097) and PC2 (*P* = 0.068). **(C)** PLS-DA permutation plot for serum samples, demonstrating model robustness with R²Y(cum) = (0, 0.91) and Q²(cum) = (0, -0.05), indicating good fit and limited overfitting. **(D)** PLS-DA permutation plot for urine samples, assessing model stability with R²Y(cum) = (0, 0.9) and Q²(cum) = (0, -0.38). **(E)** OPLS-DA permutation plot for serum samples, with R²Y(cum) = (0, 0.91) and Q²(cum) = (0, -0.63), supporting model validity. **(F)** OPLS-DA permutation plot for urine samples, with R²Y(cum) = (0, 0.9) and Q²(cum) = (0, -0.52).

Supplementary Figure 2 Spectral and quality-control evaluation of targeted bile acid metabolomics. **(A)** Representative chromatogram acquired in negative-ion MRM mode. **(B)** Representative chromatogram acquired in positive-ion MRM mode. **(C)** Representative total ion chromatogram of a standard sample. **(D)** Overlay of total ion chromatograms from QC samples.

Supplementary Figure 3 The Z-score plot of DEMs in serum **(A)** and urine **(B)** samples in untargeted metabolomics.

Supplementary Table 1 Top enriched pathways in the serum and urine untargeted metabolomics analyses.Total, number of metabolites in the pathway; Hits, number of differential metabolite hits on the pathway; Impact, the impact value for topology analysis of the metabolic pathway.
