# Supplementary material for: Integrated untargeted and targeted metabolomics identifies and validates urinary bile acid biomarkers for the diagnosis of intrahepatic cholestasis of pregnancy
Source: Front Endocrinol (Lausanne). 2026 Sep 23;17:1944962. doi: 10.3389/fendo.2026.1944962 (PMC13642224; doi:10.3389/fendo.2026.1944962)
Supplement: Supplementary file 1 [file DataSheet1.docx]

**LC-MS analysis for untargeted metabolomics analysis**

***Chromatography detection parameters***

The column (Waters, ACQUITY UPLC HSS T3 1.8 µm, 2.1 mm×100 mm) was employed. Urine samples were separated by chromatography with the column temperature at 40 ℃ and the flow rate of 0.3 mL/min. The mobile phase A consisted of water and 0.05% formic acid (CNW, Shanghai China) while the mobile phase B consisted of acetonitrile (Merck, Darmstadt Germany). The elution gradient was started initially with 95% A for 1 min, linearly decreased to 5% A at 12 min, maintained for 1.6 min, and then returned to 95% A for 2.4 min of equilibrium. The automatic injector temperature was 4 ℃, and 5 µL of aliquot of each specimen was injected into the column. Time of flight (TOF)/MS was conducted on both positive ion mode and negative ion mode.

***Mass spectrometry detection parameters***

1) Below were electrospray ionization (ESI) source conditions:

ESI+ : heater temperature, 300 ℃; sheath gas flow rate, 45 arb; aux gas flow rate, 15 arb; sweep gas flow rate, 1 arb; spray voltage, 3.0 KV; capillary temperature, 350 ℃; S-Lens RF level, 30%;

ESI- : heater temperature, 300 ℃; sheath gas flow rate, 45 arb; aux gas flow rate, 15 arb; sweep gas flow rate, 1 arb; spray voltage, 3.2 KV; capillary temperature, 350 ℃; S-Lens RF level, 60%.

2) The parameters of scan mode were set as follows:

full scan (m/z 70~1050);

data-dependent secondary mass spectrometry (dd-MS2, TopN=10);

resolution, 70,000 (primary mass spectrometry) & 17,500 (secondary mass spectrometry);

collision mode, high-energy collisional dissociation (HCD).

**LC-MS/MS analysis for targeted metabolomics analysis**

***Chromatography detection parameters***

Injection volume: 5 μL;

Flow rate: 0.45 mL/min;

Flow phase: A (0.1% formic acid-water solution), B (methanol: acetonitrile: isopropanol = 1:1:1, containing 0.1% formic acid);

Gradient elution procedures: 0 min A/B (80:20, V/V), 1 min A/B (80:20, V/V), 1.5 min A/B (62:38, V/V), 7.5 min A/B (50:50, V/V), 10 min A/B (5:95, V/V), 11 min A/B (5:95, V/V), 12 min A/B (80:20, V/V).

***Mass spectrometry detection parameters***

Curtain gas: 35 (psi);

Collision-activated dissociation (CAD) parameter: medium;

Negative/positive ion spray voltage: -4500V/+5500V;

Ion source temperature: 450 ℃; Column temperature: 45 ℃;

Spray gas (Gas1): 55 (psi); Auxiliary heating gas (Gas2): 55 (psi).

Note: The QC samples were injected and detected at regular intervals (every 10 samples) across the analytical procedure to provide a set of data that could be used to assess reproducibility.

***Multi-reaction monitoring (MRM) transition***

Metabolite quantification was performed using the Multiple Reaction Monitoring (MRM) mode of triple quadrupole mass spectrometry. In the MRM mode, the quadrupole first screens for the precursor ions (parent ions) of the target compound, eliminating ions corresponding to other molecular weight substances to preliminarily exclude interference. The precursor ions are then ionized and fragmented in the collision cell, generating multiple fragment ions. These fragment ions are subsequently filtered through the triple quadrupole to select the desired characteristic fragment ion, thereby excluding non-target ions and ensuring more precise quantification with better reproducibility. Mass spectrometric data from different samples were obtained, and the peak areas of all chromatographic peaks were integrated. Additionally, integration corrections were performed for chromatographic peaks of the same substance across different samples.

Figure. Schematic diagram of multi-reaction monitoring (MRM) transition.

**Conversion formula**

Sample metabolite concentration (ng/mL) = ***N1 * N2 * (C * V)/M***,

(***C***: concentration value (ng/mL) calculated by taking the metabolite peak area in the sample to the standard curve; ***V***: constant volume (0.2mL); ***M***: sampling volume (mL); ***N1***: conversion coefficient (10/9); ***N2***: dilution factor.)
