# Supplementary material for: Integrated untargeted and targeted metabolomics identifies and validates urinary bile acid biomarkers for the diagnosis of intrahepatic cholestasis of pregnancy
Source: Front Endocrinol (Lausanne). 2026 Sep 23;17:1944962. doi: 10.3389/fendo.2026.1944962 (PMC13642224; doi:10.3389/fendo.2026.1944962)
Supplement: Supplementary file 2 [file Table1.docx]

**Table S1**. Top enriched pathways in the serum and urine untargeted metabolomics analyses.

| **Sample type** | **Pathway** | **Total** | **Hits** | **P value** | **Impact** | **Name of hits** |
| --- | --- | --- | --- | --- | --- | --- |
| Serum | Primary bile acid biosynthesis | 46 | 5 | 0.00308 | 0.03564 | Cholic acid;  Glycochenodeoxycholate;  Taurochenodeoxycholate;  Glycocholate;  Taurocholate |
|  | Fatty acid biosynthesis | 47 | 3 | 0.08691 | 0 | Tetradecanoic acid;  Dodecanoic acid;  Decanoic acid |
|  | Valine, leucine and isoleucine biosynthesis | 8 | 1 | 0.16734 | 0 | 4-Methyl-2-oxopentanoate |
|  | Taurine and hypotaurine metabolism | 8 | 1 | 0.16734 | 0 | Taurocholate |
|  | Glycerophospholipid metabolism | 36 | 2 | 0.19414 | 0.0655 | 1-Acyl-sn-glycero-3-phosphocholine;  sn-Glycero-3-phosphocholine |
| Urine | beta-Alanine metabolism | 21 | 4 | 0.00281 | 0.05597 | 3-Hydroxypropanoate;  5,6-Dihydrouracil;  Uracil;  L-Histidine |
|  | Histidine metabolism | 16 | 3 | 0.01045 | 0.22131 | L-Glutamate;   L-Histidine;  Imidazole-4-acetate |
|  | D-Glutamine and D-glutamate metabolism | 6 | 2 | 0.01198 | 0.5 | L-Glutamate;  D-Glutamine |
|  | Primary bile acid biosynthesis | 46 | 4 | 0.04458 | 0.02587 | Cholic acid;  Glycochenodeoxycholate;  Glycocholate;  Taurocholate |
|  | Arginine biosynthesis | 14 | 2 | 0.06252 | 0.11675 | L-Glutamate;  N-Acetyl-L-glutamate |

**Note**:

Total, number of metabolites in the pathway;

Hits, number of DEMs mapped on the pathway;

Impact, pathway topology impact value.
